# Characteristics of resistivity variation in deep granite and in-situ detection applications

**DOI:** 10.1038/s41598-024-56695-x

**Published:** 2024-03-13

**Authors:** Zhen Fu, Yuezheng Zhang, Hongguang Ji, Chunrui Zhang, Dongsheng Chen, Yuan Qin

**Affiliations:** 1 Beijing Key Laboratory of Urban Underground Space Engineering , University of Science and Technology Beijing, Beijing, 100083 China; 2 National Engineering Research Center of Deep Shaft Construction, Beijing, 100013 China

**Keywords:** Civil engineering, Geophysics, Hydrogeology

## Abstract

During the construction of deep vertical shafts, water inrush and flooding accidents are prone to occur, which seriously affect construction safety. Accurately determining the groundwater conditions is a prerequisite for effectively controlling water hazards and conducting risk management. In order to ensure the accuracy of the resistivity method in deep vertical well water exploration construction, a combination of indoor rock physics, mechanical testing, and on-site engineering measurements was used to analyze the influencing factors of granite resistivity. The corresponding relationship between resistivity and formation integrity was revealed, and water exploration experiments were conducted in the working face of deep underground mines. The results show that: (1) Rock resistivity is influenced by metallic minerals, saturation, temperature, ion content of fracture water, and joints. Regarding deep subsurface detection issues, the main factors affecting the detection results are water content and rock integrity. (2) During the loading process, rock resistivity exhibits significant stage response characteristics, which are closely related to rock integrity and damage accumulation. (3) A degradation model for aquifer zoning based on resistivity benchmark line was established. When the formation resistivity is higher than the benchmark line, it indicates a well-integrated formation with low water content. (4) Resistivity cloud maps and zoning degradation models can be used to visually determine and evaluate the occurrence status of formations and the effectiveness of grouting.

## Introduction

**Figure 1:**
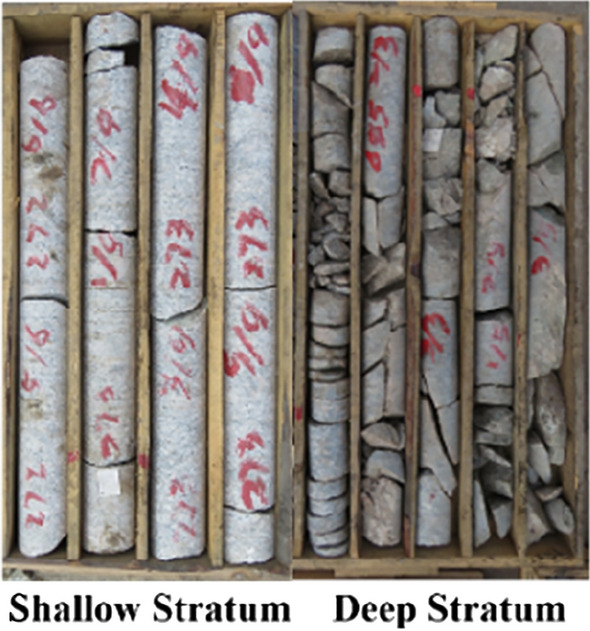
Comparison chart of core samples from shallow and deep stratum.

With the continuous growth in the demand for deep underground resource development, vertical shaft depths have exceeded 1500m, leading to increasingly prominent challenges in deep geological conditions^1–4^. By comparing the core samples from engineering exploration boreholes (Fig. 1), it is observed that shallow formations have relatively low stress levels, resulting in intact core samples with fewer joint fractures. However, as depth and crustal stress levels increase, the integrity of core samples gradually decreases, accompanied by the occurrence of discing phenomenon. When the depth exceeds 1600 m, core samples mainly consist of fragmented blocks, with highly developed joint fractures. The elevated stress levels in deep formations make rocks more prone to plastic deformation, further complicating the engineering geological conditions for deep construction projects.

In deep geological formations, under the influence of complex geological conditions, disasters such as collapses, rock bursts, water influx, and sudden water outbursts often occur after excavation disturbances. Among these disasters, water influx is particularly significant. It can result in project delays and increased construction costs in less severe cases, while in more severe cases, it can lead to well flooding and abandonment. Both research and engineering practice have consistently shown that water hazards have become the primary risk in the construction of deep well projects^5–8^. Therefore, improving the accuracy and reliability of engineering geological and hydrogeological investigations within the vertical shaft project area is a prerequisite for scientifically assessing water inflow risks and effectively managing water disaster.

For rocks, electrical resistivity is an important parameter that can explain the composition and structural changes of minerals and rocks in the deep Earth^9,10^. Zhang et al.^11^, Chen et al.^12^ and others have found through experimental research that the electrical resistivity of rocks decreases with increasing water content. Kelvin et al.^13^ established a modeling method for the variation of electrical properties of saturated sandstone with pressure by studying the correspondence between elastic modulus, electrical resistivity, and stress under different stress states. The model results showed good agreement with laboratory results. Shmulik^14^ through designed experimental studies, found that the volumetric water content of soil is the main factor affecting soil conductivity and that it has a strong dependence on water content. Bai et al.^15^, Li et al.^16^ and other researchers have found a good correlation between mineral composition and soil electrical conductivity, with metallic minerals enhancing electrical conductivity, and the electrical conductivity of red soil increasing with increasing water content, saturation, and dry density. Zhang et al.^17^ found that electrical resistivity decreases with increasing salinity and increasing saturation. Many scholars Dong et al.^18^, Sumi et al.^19^, Corwin et al.^20^, and Mohamed^21^ have conducted a series of studies confirming the direct relationship between rock resistivity and degree of mineralization. The quality of rock mass, water content, and hydrochemical characteristics in deep geological formations all have an impact on their electrical resistivity, resulting in significant differences in resistivity. Therefore, the resistivity method can be used to evaluate the state of stratigraphic condition based on these differences.

In practice, traditional water exploration methods face many difficulties due to the complex working conditions of vertical well faces. However, electrical resistivity is closely related to the degree of rock fragmentation and water content. Therefore, this paper analyzes the influencing factors of granite resistivity by combining indoor rock physics (mineral composition, water content, joints and temperature), cyclic loading and unloading mechanical tests and on-site working face cross-hole resistivity scanning. It reveals the corresponding relationship between resistivity and stratigraphic conditions in deep granite formations. The resistivity method is then applied for field measurements in mining areas, providing a scientific basis for safe construction in mines.

## Experimental study on the variation characteristics of electrical resistivity

**Figure 2:**
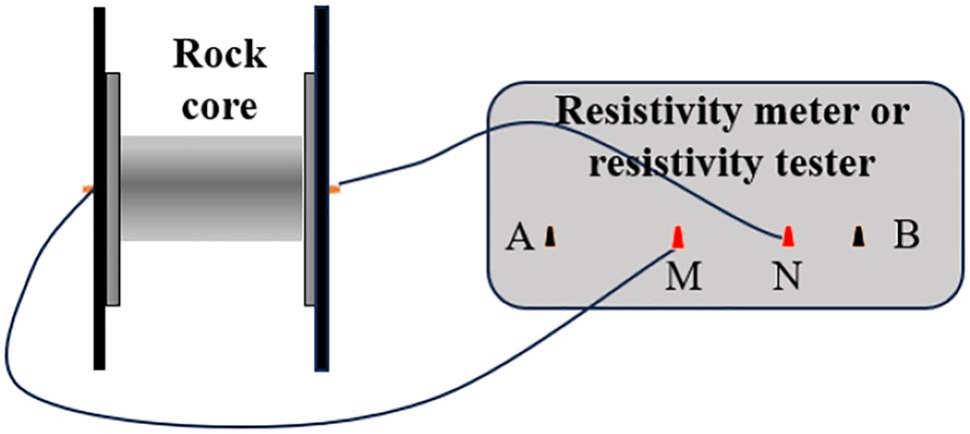
Rock resistivity testing device.

Indoor rock resistivity measurements are conducted using the dipole method^22^. The electrodes are symmetrically arranged on both sides of the rock. By measuring the resistance value R, length L, and cross-sectional area A of the rock, the resistivity of the entire core can be calculated using the resistivity formula (1). The resistivity is inversely proportional to the length of the core and directly proportional to the resistance and cross-sectional area. The measurement method is illustrated in Fig. 2. In the mining face, rock cores are taken and processed into standard cylindrical samples with a diameter of 50mm and a height of 100mm. The resistivity of the granite is tested using the EDYC-2 rock sample electrical testing instrument. The physical and mechanical test samples designed in this paper are shown in Table 1.

1 $$\rho = R\frac{A}{L}$$

**Table 1:** Test sample data information.

Sample source	Laboratory naming	Sample depth/m	Design test block	Density/kg·m^−3^	Wave velocity/km·s^−1^	Essential component	Test contents
Working face of stope	Pyrite-Bearing granite	1000	Figs. 3, 5	\	\	Quartz, feldspar, pyrite, etc	Electron microscope scanning; resistivity test
Shaft tunneling working face	Black biotite monzonitic granite	903	Figs. 3, 4, 5, 6	\	\	Quartz, feldspar, biotite, etc	XRD, scanning electron microscopy, resistivity test
			0°; 30°; 45°; 60°; 90°	2.62; 2.85; 2.62; 2.61; 2.60	5.32	Quartz, feldspar, biotite, etc	Resistivity, thermal resistance rate test
					5.10		
					5.81		
					5.56		
					5.32		
			L1	2.63	5.95	Quartz, feldspar, biotite, etc	Cyclic loading and unloading resistivity test
			M2	2.62	5.81	Quartz, feldspar, biotite, etc	Cyclic loading and unloading resistivity test

**Figure 3:**
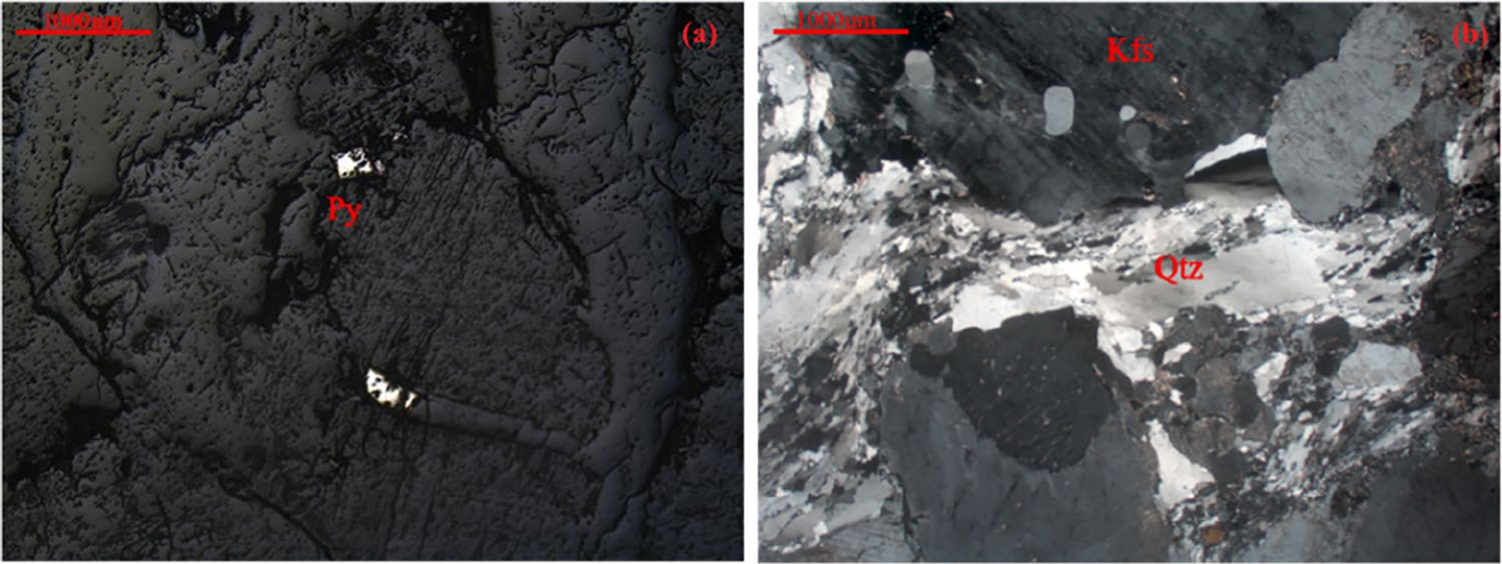
Polarized light microscope scanning results.

**Figure 4:**
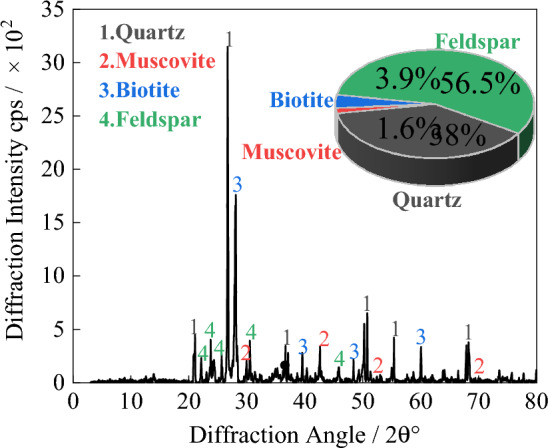
XRD scanning results.

**Figure 5:**
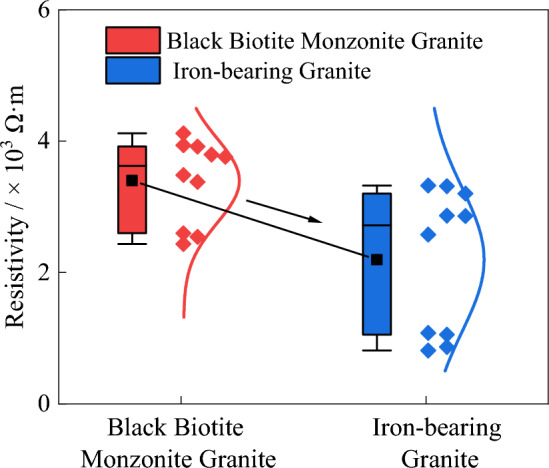
The resistivity comparison of difference granites.

**Figure 6:**
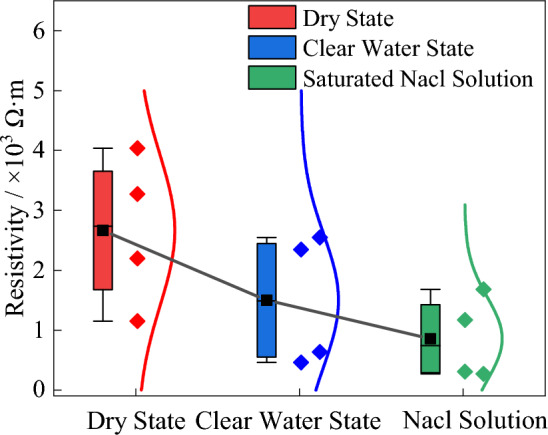
The variation of black biotite monzonitic granite resistivity in different environments.

In the equation, *R* is the resistance, *A* is the cross-sectional area and *L* is the length.

### Characterization of the effect of mineral composition

The composition of the granite was analyzed using polarized microscope and X-ray diffraction (XRD) tests. The obtained images are shown in Figs. 3 and 4.

The major components of granite are feldspar and quartz, with the combined content of these two minerals accounting for over 94.5% of the granite composition. The remaining approximately 5.5% consists of other minerals such as biotite, mica, and metallic minerals.

The electrical resistivity of different components in granite varies to some extent. The wide range of resistivity in granite is primarily due to the different presence states of various minerals. However, the variation range of resistivity for metallic minerals is significantly lower compared to minerals such as feldspar, quartz, and mica. Consequently, as the content of metallic minerals increases in the rock, the resistivity tends to decrease.

Samples of black biotite granite and metal-bearing mineral granite were collected from the working face of gas well mining and shaft tunnel excavation in Xincheng Gold Mine, Yantai City, Shandong Province, and processed into core samples with a diameter of 50 mm and a height of 100 mm. The metallic mineral content in the granite from the quarry face was relatively high. The resistivity of the black biotite granite and metallic mineral-bearing granite was tested, and the test results were plotted in a box plot, as shown in Fig. 5. The average resistivity of the black biotite granite was 3.39 kΩ·m, while the average resistivity of the iron-bearing granite was 2.20 kΩ·m. The presence of metallic minerals caused an average decrease in resistivity of 35.10%. However, since the majority of granite samples have relatively low metallic mineral content, the impact of this factor on the detection results is relatively minor.

### Characterization of the effect of water content

The resistivity of rock is closely related to water content and ion concentration^23^. The granite samples were placed in a drying furnace (drying temperature of 60 °C), distilled water and saturated NaCl solution for 48 h. During the experiment, the rock was weighed every 6 h. After the weight of the sample is stable, it is considered that the rock reaches a drier or water-bearing state than the natural state. At this point, the resistivity is measured and the results are plotted in a box diagram, as shown in Fig. 6.

The electrical resistivity of rocks decreases with increasing water content and concentration of conductive ions. In the dry state, where there are no conductive ions present internally, only the conductive minerals contribute to the electrical conductivity, resulting in the highest resistivity. In the fully saturated state, small primary cracks or fissures within the rock provide pathways for conduction, leading to a decrease in resistivity. In the case of saturated NaCl solution, the presence of conductive ions allows for free migration through the water-conducting pathways formed by the primary cracks, resulting in even lower resistivity compared to the first two states.

Comparing the resistivity in the dry state, the resistivity decreases by 43.82% in the fully saturated state and by 67.84% in the saturated NaCl solution. Therefore, this significant decrease in resistivity due to the presence of water and conductive ions highlights the importance of these factors in deep geological exploration.

### Effects of joint on rock characteristics

**Figure 7:**
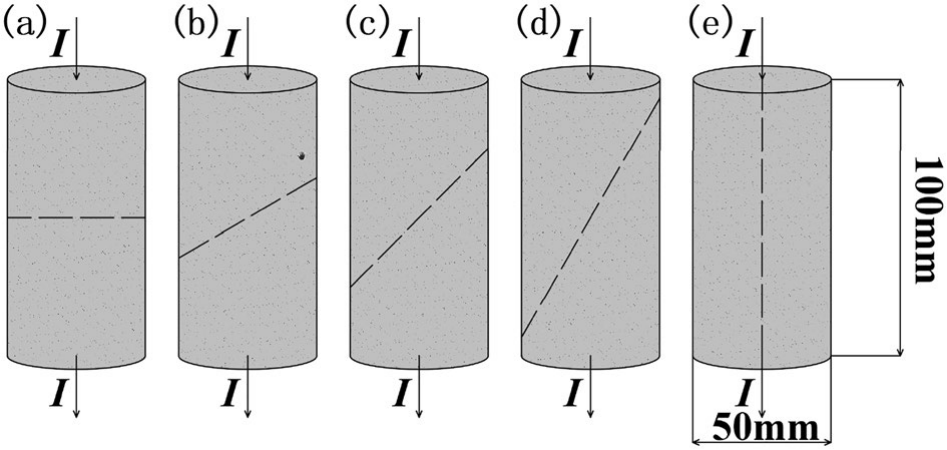
Jointed biotite monzonitic granite rock processing schematic.

**Figure 8:**
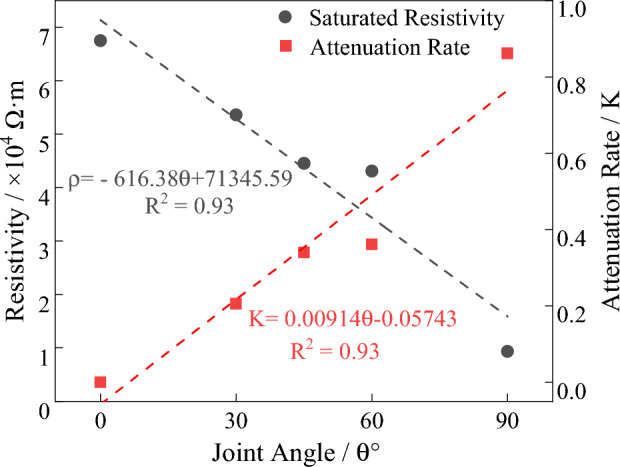
The variation of resistivity of jointed biotite monzonitic granite with angle.

Rock as a natural heterogeneous material, contains numerous inherent defects, and its electrical resistivity is closely related to joints and fractures. To verify the relationship between electrical resistivity and rock joints, granite rock samples with different angles of joints were processed, as shown in Fig. 7. The variations in electrical resistivity were measured and are illustrated in Fig. 8.

In the saturated state, the electrical resistivity decreases gradually with increasing joint angle, following a linear decreasing trend. As the joint angle increases, through-going joints become more parallel to the direction of electrical current, improving the conductivity conditions and resulting in a decrease in resistivity. Therefore, when there are water-conducting channels present in the subsurface being explored, the resistivity will exhibit a low-resistivity discontinuity. The mathematical model describing the relationship between resistivity and joint angle variation is given by Eq. (2).

The electrical resistivity angle attenuation rate, denoted as K, is defined by the Eq. (3). The attenuation rate increases gradually with increasing angle and exhibits a linear increasing trend. The mathematical model relating the joint angle to the attenuation rate is described by Eq. (4).

2 $$\rho = - 616.38\theta + 71345.56,\,\,\,\,{\text{R}}^{{2}} = 0.{93}$$

3 $$K = \frac{{\rho_{\theta = 0} - \rho_{{\theta_{i} }} }}{{\rho_{\theta = 0} }}$$

4 $${\text{K}} = 0.00914\theta^{{}} - 0.05743,\,\,\,{\text{R}}^{{2}} = 0.{93}$$

In the equation, $K$ represents the angle attenuation rate, $\rho_{\theta = 0}$ represents the resistivity when the joint angle is 0, and $\rho_{{\theta_{i} }}$ represents the resistivity at the given angle $\theta_{i}$.

### Effects of ambient temperature

**Figure 9:**
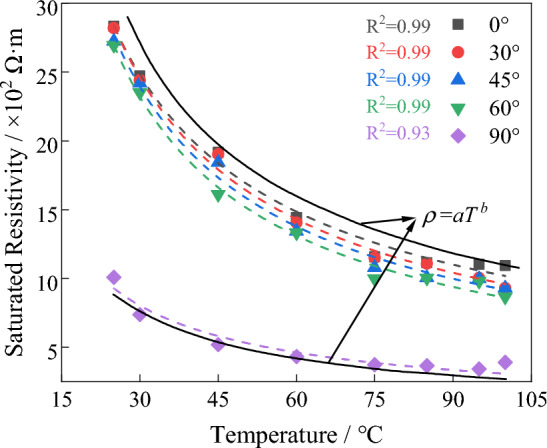
Variation of resistivity of jointed biotite monzonitic granite with temperature.

**Figure 10:**
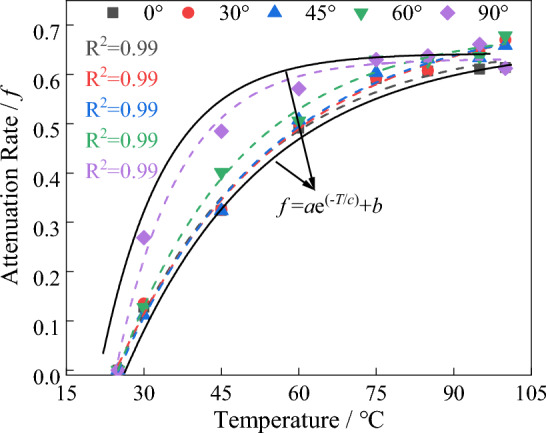
Variation of resistivity attenuation rate of jointed biotite monzonitic granite with temperature.

Temperature plays a role in influencing the migration rate of ions and thus affects the changes in electrical resistivity of water-bearing rocks^24,25^. Considering the actual temperature during the detection process, a constant-temperature water bath was used to heat granite samples with different joint angles for 48 h. The temperature levels used were: 25 °C, 30 °C, 45 °C, 60 °C, 75 °C, 85 °C, 95 °C, and 99.5 °C (In practice, the water temperature is controlled at 99.5 °C ± 0.2 °C to prevent water from boiling). Immediately after heating, the electrical resistivity of the samples was measured, as shown in Fig. 9. As the temperature increases, the migration rate of conductive ions in the fracture water accelerates, leading to a gradual decrease in electrical resistivity, following an exponential decreasing trend. The relationship between temperature and electrical resistivity can be fitted using Eq. (5). The temperature attenuation rate of rock resistivity is defined, and its calculation formula is given by Eq. (6). The attenuation rate increases gradually with increasing temperature, as shown in Fig. 10.

5 $$\rho = aT^{{\text{b}}}$$

6 $$f = \frac{{\rho_{T = 25} - \rho_{{T_{i} }} }}{{\rho_{T = 25} }}$$

7 $$f = a{\text{e}}^{{{(} - {\text{T/c)}}}} + b$$

In the equation: $f$ represents the temperature attenuation rate; $\rho_{T = 25}$ represents the resistivity at an initial temperature of 25 ℃; $\rho_{{T_{i} }}$ represents the resistivity at the given temperature $T_{i}$; a and b are coefficients.

Based on the physical experiments regarding the resistivity variation characteristics mentioned above, it can be observed that the resistivity of granite is closely related to mineral composition, water content, joint fractures, and ambient temperature. However, for deep subsurface exploration, the content of metallic minerals is extremely low, resulting in minimal impact on the detection results. Additionally, the temperature in deep subsurface environments remains relatively constant. Therefore, the important factors influencing the detection results are the formation's water content and joint fractures.

To further explore the variation patterns of resistivity in rocks under different loading conditions and integrity levels, a graded cyclic loading and unloading test was designed for granite. This study aims to investigate the relationship between rock integrity and resistivity.

## The characteristics of resistivity variation under loading conditions

### Experimental protocol

**Figure 11:**
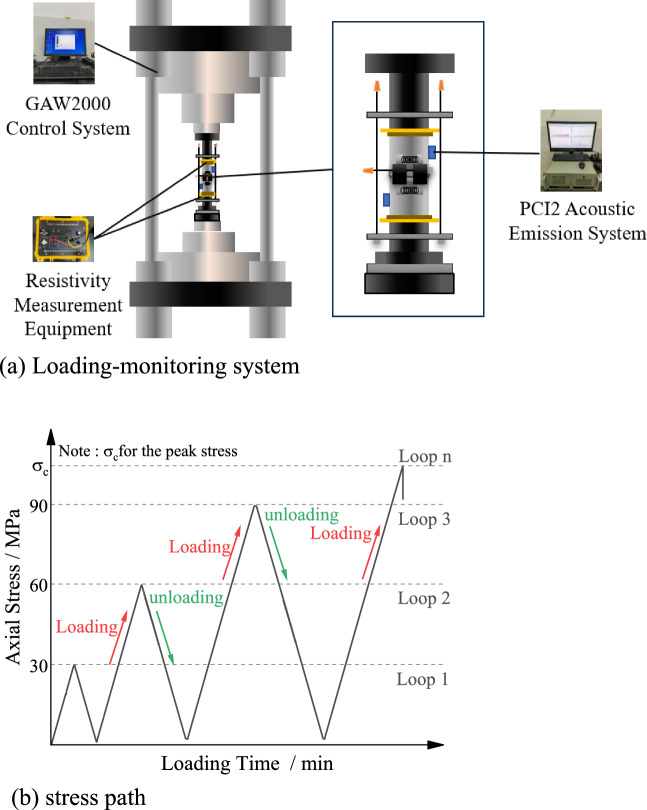
Loading-monitoring system and stress path.

For studying the characteristics of resistivity variation during the degradation process under loading, the GAW-2000 rigid testing machine shown in Fig. 11a was used to perform the loading and unloading tests. Strain gauges were employed to measure the axial and circumferential strains of the rock samples. M and N poles for resistivity testing were installed on both sides of the rock sample, and insulation pads were added to prevent electrical signal interference. The EDYC-2 electrical analyzer was used to collect resistivity data. Simultaneously, the PCI-2 type acoustic emission equipment was used to monitor acoustic emission signals, which provided real-time information on the degradation of the rock sample.

The loading was conducted in a stepwise manner, incrementing (or decrementing) the axial load. The maximum load for each cycle was increased by 30 kN compared to the previous cycle. The loading rate was set to 300 N/s, while the unloading rate was also set to 300 N/s. The stress paths during loading and unloading are illustrated in Fig. 11b.

### The characteristics of resistivity variation during the cyclic loading process

**Figure 12:**
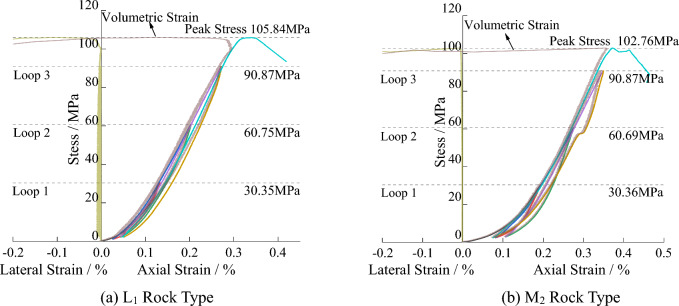
The stress–strain curve of the specimen.

To avoid errors caused by the rock samples, the longitudinal wave velocities of the two rock samples were measured before the start of the experiment. Rock samples with similar longitudinal wave velocities were selected for the test. The average compressive strength of the rock samples was 104.30 MPa, and the average axial peak strain was 0.37. During the loading and unloading process, the presence of inherent cracks, joints, and small pores within the granite leads to the rock samples deviating from the behavior of an ideal elastic material. The unloading and loading curves did not overlap and exhibited a significant "hysteresis" phenomenon^26^, as shown in Fig. 12.

**Figure 13:**
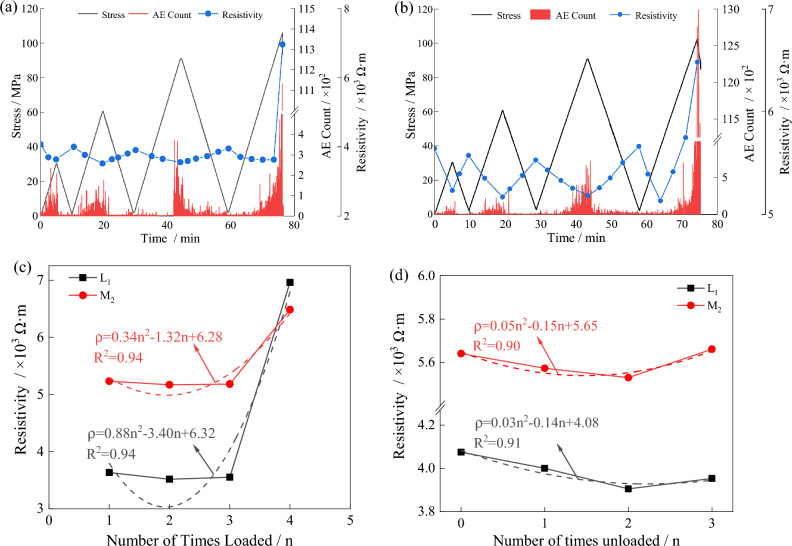
The variation of resistivity with stress level.

The generation of hysteresis loops is accompanied by changes in the internal structure of granite, which lead to corresponding variations in resistivity under different stress conditions. The resistivity variation with stress during the loading process is shown in Fig. 13a and b, while the resistivity variation during complete loading and unloading of the specimen is shown in Fig. 13c and d. The specific relationship between resistivity and stress variation is as follows^27,28^:

During the loading and unloading process, acoustic emission signals are constantly generated. As each stage reaches its peak load, the acoustic emission signals become more active, and the count of acoustic emission signals during the loading process is much higher than during the unloading process. When the peak stress is reached, a large number of acoustic emission signals are generated. The resistivity follows a general trend of decreasing and then increasing during the loading process, reaching its maximum value at the point of rock failure.

In the initial loading process, the resistivity gradually decreases. There are many inherent microcracks and pores inside the rock, which rapidly close under external load, allowing mineral particles to come into full contact, resulting in a gradual decrease in resistivity. After the start of unloading, the resistivity gradually recovers but remains lower than the initial unloaded state. But during the initial loading process, the stress has reached the bearing strength (Stress level in compaction stage) of micro cracks and pores. After unloading, the deformation in the elastic range is restored, and the deformation reaching the bearing strength cannot be completely restored. Therefore, the rock resistivity is lower than that before loading.

During the second loading process, the resistivity still exhibits a gradual decreasing trend initially. Compared to the previous cycle, the resistivity decreases even lower at the peak load of this stage. During the 0 MPa to 30.35 MPa stage, the rock undergoes further compaction, resulting in a decrease in resistivity. When the load is unloaded from 30.35 MPa to 0 MPa, the resistivity gradually recovers. However, this cycle generates irrecoverable residual deformation that is greater than the previous cycle. Therefore, the resistivity is lower after complete unloading compared to the previous cycle.

In the third loading process, the resistivity initially shows a gradual decrease, but the resistivity at the peak of this cycle is higher than the previous cycle. When the external load exceeds the peak value of the previous cycle, a larger number of newly formed cracks are generated. These numerous new cracks lead to a decrease in compactness, resulting in a higher resistivity at the peak of this cycle compared to the previous cycle. When the load is unloaded from 60.75 MPa to 0 MPa, the resistivity gradually recovers. However, due to the generation of a large number of new cracks, the resistivity of the rock is higher than the previous cycle.

During loading until rock failure, the resistivity still shows a gradual decreasing trend. However, during the previous cycle, a significant number of new cracks accumulate inside the rock. Therefore, when the stress continues to increase, the resistivity gradually begins to increase. When macroscopic through-cracks form on the surface of the rock, the resistivity experiences a sudden increase.

### Synergistic relationship between rock damage process and resistivity

**Figure 14:**
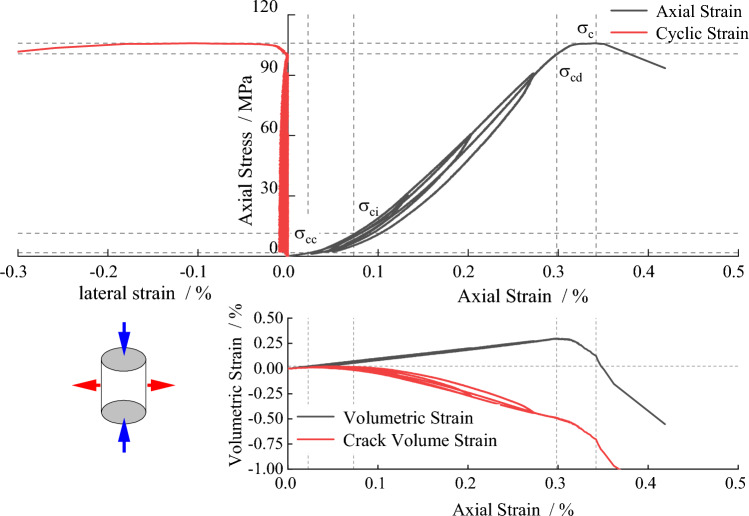
Cyclic loading and unloading stage division diagram of specimen L_1_.

The deterioration of rocks under loading corresponds to the evolution of internal cracks. According to the change rule of resistivity in the process of cyclic loading, it can be inferred that the evolution of cracks is closely related to resistivity. To analyze the synergistic relationship between rock crack damage and resistivity variation, the crack volume strain can be calculated using the crack volume strain method^29^. The calculation formulas are shown in Eqs. (8), (9) and (10), and the phase division diagram of crack volume strain for the L1 specimen is plotted in Fig. 14.

Calculation method for overall volumetric strain under conventional uniaxial loading:

8 $$\varepsilon_{v} = \varepsilon_{1} + 2\varepsilon_{3}$$

In the equation, $\sigma_{1}$ represents volumetric strain, $\varepsilon_{1}$ represents axial strain, and $\varepsilon_{3}$ represents radial strain.

The volumetric strain calculated during the elastic stage is:

9 $$\varepsilon_{v}^{e} = \frac{1 - 2v}{E}\sigma_{1}$$

In the equation, $\varepsilon_{v}^{e}$ represents elastic volumetric strain, $v$ represents Poisson's ratio is calculated by the linear elastic stage of the stress–strain curve, E is calculated by the linear elastic stage of the stress–strain curve, and $\sigma_{1}$ a represents axial stress.

The calculation of crack volume strain using volumetric strain and elastic volumetric strain yields:

10 $$\varepsilon_{v}^{c} = \varepsilon_{v} - \frac{1 - 2v}{E}\sigma_{1}$$

During the cyclic loading and unloading process, the development of pre-peak cracks can be divided into four stages: compaction stage, elastic deformation stage, stable crack propagation stage, and unstable propagation stage. Combining Figs. 13 and 14, it can be observed that during the first three loading cycles, the stress level did not reach the threshold for stable crack development. As a result, the range of resistivity variation was small, with resistivity decreasing from 3.63 kΩ·m to 3.55 kΩ·m. However, when the stress level exceeded the threshold for stable crack development, the resistivity increased to 5.56 kΩ·m, indicating a significant increase in resistivity variation.

Using the acoustic emission damage variable, the resistivity characteristics during the damage deterioration process of the rock sample can be described. According to the method for calculating damage parameters using acoustic emission described in reference^30^, the relationship between the damage parameter D and the cumulative energy value for the rock is given by Eq. (11):

11 $$D = \frac{{N_{t} }}{{N_{m} }}$$

In Eq. (11), D represents the acoustic emission damage variable, *N*_*t*_ represents cumulative energy value corresponding to $\sigma_{i}$, and *N*_*m*_ represents the cumulative energy value at complete failure.

**Figure 15:**
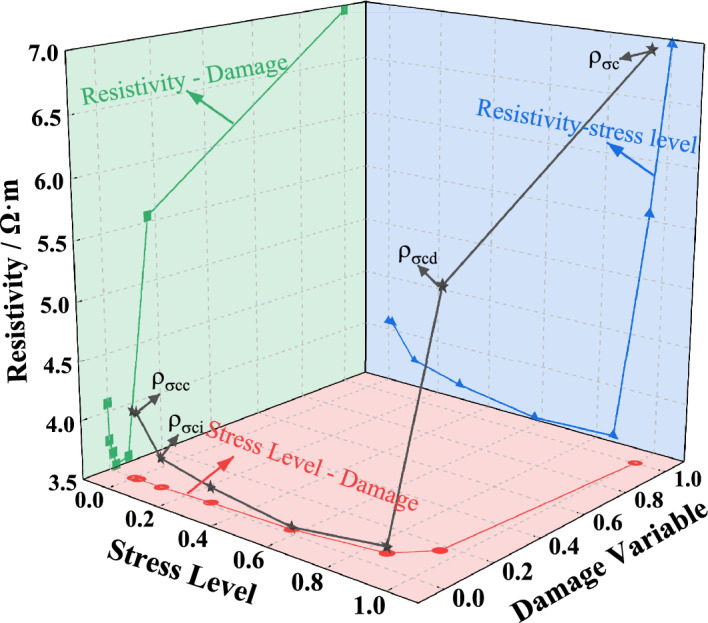
The relationship among stress level, damage variable and resistivity of L_1_ specimen during loading process. The green line is the relationship between the resistivity and the damage variable; the red line is the relationship between stress level and damage variable; The blue line is the relationship between the stress level and the resistivity; The black line is the resistivity change during the loading process.

Using the acoustic emission damage calculation formula, the damage deterioration of the L1 specimen during the cyclic loading and unloading process can be computed. Simultaneously, the corresponding resistivity changes are statistically analyzed to establish the relationship between the damage variable, resistivity, and different stress levels. The results are plotted in Fig. 15. At a stress level of 60% of the peak stress, within the threshold of stable crack propagation, the damage variable ranges from 0 to 0.02, and the resistivity gradually decreases. As the stress level gradually increases to 60% of the peak stress, the damage variable increases to 0.17, and the resistivity begins to rise. When the stress level reaches the peak stress, the damage variable reaches 1, and the resistivity reaches its maximum value.

In conclusion, resistivity exhibits distinct stage response characteristics during the rock deterioration process, closely related to rock integrity. Under dry conditions, from the compaction stage to the elastic stage, resistivity shows a concave downward trend, with a gradually slowing rate of decrease. During the stable crack propagation stage, resistivity transitions from a decreasing trend to an increasing trend with the generation of cracks. From the unstable crack propagation stage to the peak stress stage, resistivity continues to rise, accompanied by a high-resistivity mutation as the rock undergoes failure.

**Figure 16:**
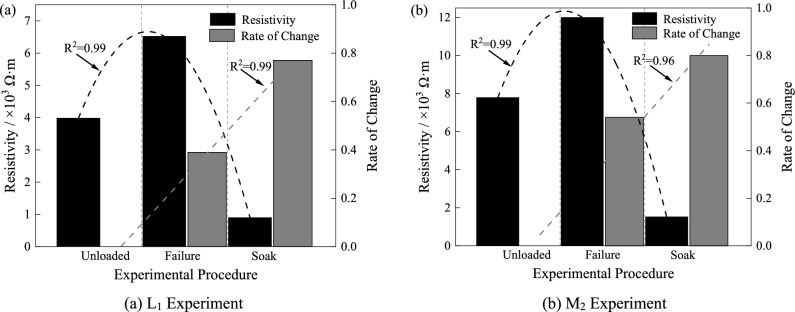
Comparison of resistivity before and after rock test.

After immersing the rock specimens that experienced loading-induced failure in water for 48 h, their resistivity was measured and plotted in Fig. 16. Taking specimen M2 as an example, the resistivity is highest at the point of failure, followed by the resistivity of the intact rock, and the resistivity of the water-soaked rock after failure is the lowest. Compared to the intact rock, the increase and decrease in resistivity after failure and water immersion are 54% and 80%, respectively. The integrity of the rock and its moisture content play a decisive role in resistivity changes. Specifically, in dry fractured formations, a high-resistivity mutation occurs, while in the presence of water, a low-resistivity mutation is observed.

## Deterioration model for stratigraphic zoning based on resistivity characteristics differences

### Stratigraphic zoning deterioration model

**Figure 17:**
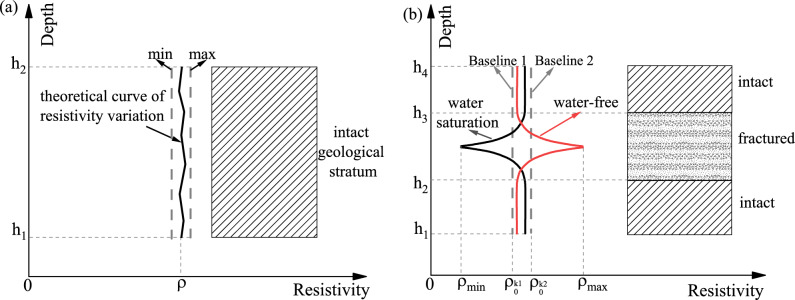
Stratigraphic zoning deterioration model diagram.

Based on the aforementioned physical and mechanical experiments, it is evident that the resistivity of rock formations in their in-situ state corresponds to their water content and integrity. According to the method proposed by E. I. Shemyaki^31^ for determining the resistivity baseline as a reference value for fractured formations, further definitions can be made. Under formation water conditions, the regions above a certain resistivity value are considered relatively intact with low water content, while the regions below that resistivity value are considered relatively fractured with high water content. The calculation method for the resistivity baseline is provided in Eq. (12), and the stratigraphic zoning deterioration model is established as shown in Fig. 17.

12 $$\left. \begin{gathered} \rho_{0}^{{\text{(k)}}} = \frac{1}{{N_{k} }}\sum\limits_{i = 1}^{{N_{k} }} {\rho_{k} \left( {x_{i} } \right)} \hfill \\ 0 < x_{i} \le L;k = 1,2, \cdot \cdot \cdot ,n \hfill \\ \end{gathered} \right\}.$$

In the equation: $k$-Borehole number; $i$-Measurement point number; $\rho_{0}^{\left( k \right)}$-Resistivity baseline value; $\rho_{k} \left( {x_{i} } \right)$-Borehole resistivity distribution function, $x_{i}$-Measurement point location; $L$-Borehole depth.

1. Intact homogeneous strata: The resistivity remains relatively constant, and the resistivity of any unit within the formation is approximately equal.
2. Intact-Fractured Water-Bearing-Intact strata: There is a significant drop in resistivity. Due to good water conductivity in the fractured strata, the likelihood of water presence is high.
3. Intact-Fractured Non-Water-Bearing-Intact: There is a sharp rise in resistivity, indicating a non-water-bearing fractured zone. The region above the resistivity baseline represents the fractured strata, while the region below represents the intact strata, consistent with the theory presented in reference^31^.

### Forward modeling simulation

**Figure 18:**
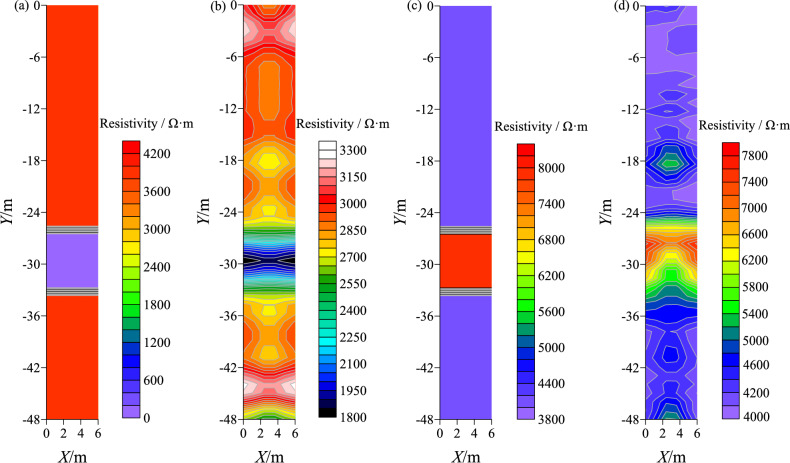
Fracture zone model and inversion.

Construct water-bearing and non-water-bearing fractured zone models for Fig. 18a and c. The borehole spacing in the model is 6 m, with a depth of 48 m. The background resistivity is 4000 Ω·m, the resistivity of the water-bearing fractured zone is 20 Ω·m, and the resistivity of the non-water-bearing fractured zone is 8000 Ω·m. The X-axis represents the borehole spacing, and the Y-axis represents the depth. Figure 18b and d show the inversion results of the model, with low resistivity anomalies and high resistivity anomalies identified in the fractured strata.

The further confirmation of the forward modeling research results indicates that the changes in Resistivity profiling and the degradation zone model can effectively and comprehensively determine the presence of fractured zones or water-rich zones between boreholes. It can also identify the distribution range and development characteristics of these anomalous bodies.

## Field experiments on stratigraphic zoning using resistivity-based degradation models

### Engineering background

The deep shaft under construction in Xiling mining area of Yantai City, Shandong Province is selected. The design depth of the shaft is more than 1500 m, and the net diameter of the section is about 10 m. In order to better assess the engineering quality of the rock mass and provide targeted guidance for construction design, the rock mass has been divided into four quality grades: intact, relatively intact, fractured, and heavily fractured, based on the core characteristics. This classification is shown in Table 2. When the wellbore crosses fractured or heavily fractured formations, the excavation work must be stopped, and on-site water exploration work should be conducted. The statistical results of the core samples indicate that near the depth of -1000 m during well construction, the working face encounters fractured and heavily fractured formations, causing significant challenges to the construction process.

**Table 2:** Rock quality classification.

The grading of rock quality	Basic qualitative characteristics of rock mass	Rock core image	Rock lithology of the strata below the working face
Intact	The rock is relatively intact, hard in nature, and the rock core exhibits a columnar shape		
Relatively intact	The rock is relatively intact, moderately hard, with moderate development of fractures within the rock mass. The rock core exhibits both long and short columnar shapes		
Fragmented	The rock mass is fragmented, with a dense distribution of fractures within the rock mass. The rock core appears in fragmented form		Rock core below the – 1000 m working face
Highly fragmented	The rock mass is highly fractured, with extremely dense distribution of fractures within the rock mass. The rock core appears angular and fragmented, resembling debris or rubble		Rock core below the – 1000 m working face

### Well logging methods

**Figure 19:**
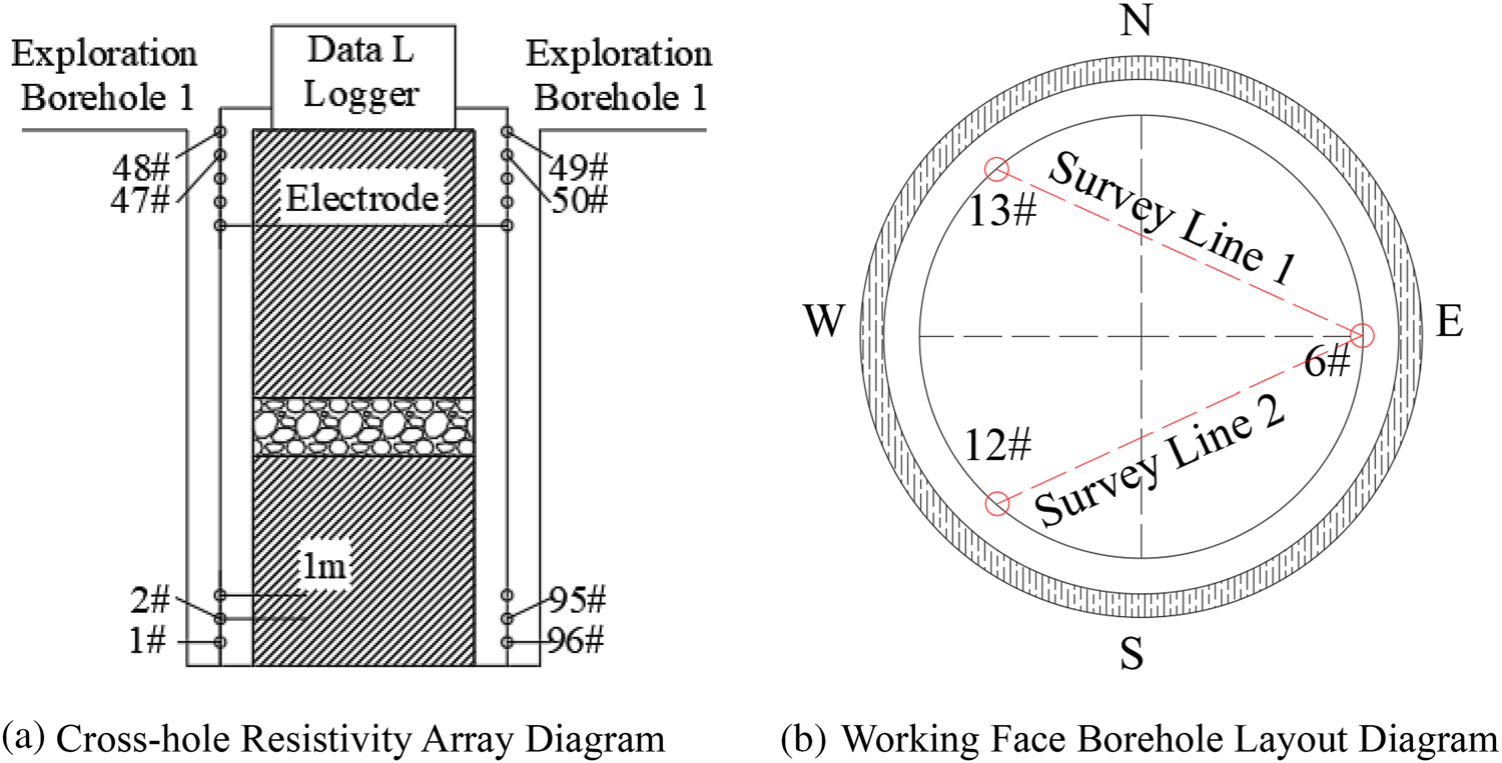
Cross-hole resistivity hole arrangement method.

According to the layout of the onsite water exploration boreholes, boreholes 6#, 12#, and 13# were selected at the working face as geophysical exploration boreholes. Among them, boreholes 6# and 13# have a depth of 100 m and a spacing of approximately 9 m. They are arranged as profile 1 for pre-grouting detection. Boreholes 6# and 12# have a depth of 100 m and a spacing of approximately 9 m. They are arranged as profile 2 for post-grouting detection. The layout of the profiles is shown in Fig. 19.

### Resistivity tomography analysis

**Figure 20:**
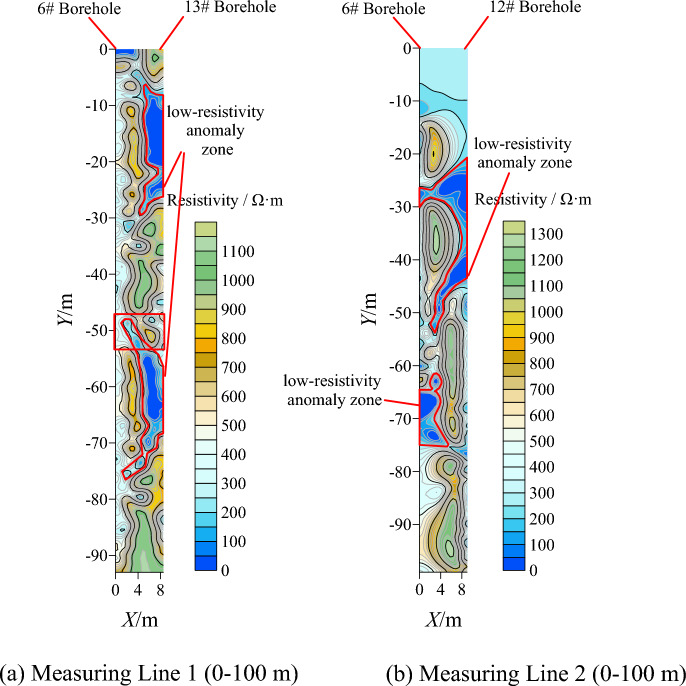
Working face resistivity cloud map.

The resistivity detection results from the working face are plotted in Fig. 20. In the figure, different colored contour lines represent the magnitude of the formation resistivity values. The higher the numerical value, the greater the formation resistivity, and vice versa.

Figure 20a shows the resistivity inversion profile of the entire profile of Line 1. Overall, within the depth range of 0–100 m, there are multiple low-resistivity anomaly areas in the formation. The low-resistivity abrupt change areas are more pronounced in the ranges of 10–30 m, 48–52 m, and 52–70 m, and there is interconnection between the boreholes. It can be inferred that the integrity of the rock formations between the two boreholes is poor, with well-developed rock joints and fractures, indicating the presence of interconnected aquifer layers. Figure 20b shows the depth profile of Line 2 within the 0–100 m range after grouting. Comparing Fig. 20a and b, it is evident that the low-resistivity abrupt change areas on the resistivity profile are significantly reduced after grouting. The resistivity mapping allows for a visual assessment of the grouting effectiveness between boreholes. Single water exploration boreholes can only observe water conditions but cannot determine the diffusion of grout between the formations or the effectiveness of the grouting work.

**Figure 21:**
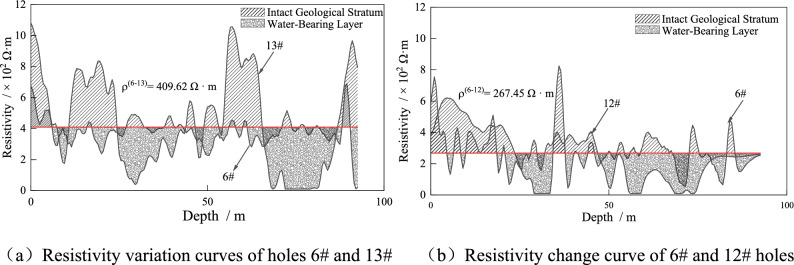
The resistivity reference value rule diagram.

According to Eq. (12), the reference resistivity values for fractured zones are calculated as shown in Fig. 21. In the presence of water, formations above the reference resistivity values indicate relatively intact formations with low water content, while formations below the reference resistivity values indicate fractured formations with poor integrity and high water content. When the resistivity values of both boreholes are below the reference resistivity values, the possibility of interconnected water conduits between the boreholes increases.

**Figure 22:**
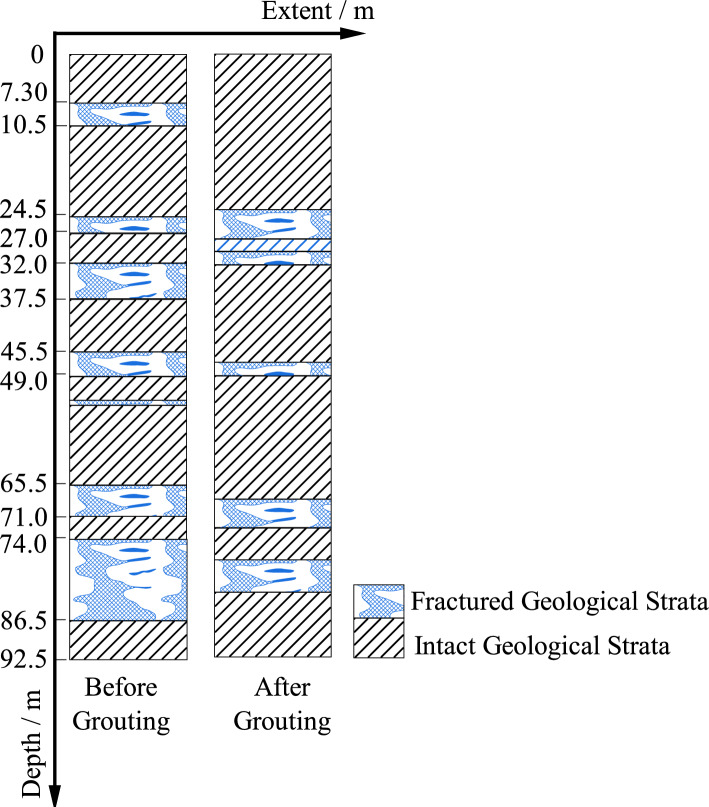
Stratum partition fracture distribution.

A degradation model for fractured aquifer zones with different thicknesses is established, as shown in Fig. 22, which provides a visual observation of the degradation distribution of the formations in front of the working face before and after grouting. Comparing the distribution of fractured aquifer zones before and after grouting, it is evident that the distribution of water-bearing fractured zones in the formations significantly reduces after grouting. The area of fractured aquifer zones before grouting is 303.3 m^2^, while after grouting, it is reduced to 157.5 m^2^, representing a 48.07% reduction in the area of fractured zones after one round of grouting. The application of the degradation model for zone classification allows for a better evaluation of the grouting effectiveness.

The practical application of resistivity method in mining sites has shown the following advantages compared to traditional water exploration methods:

1. The resistivity method provides a visual understanding and identification of the overall condition of the underlying formations below the working face through resistivity mapping. This breaks away from the traditional approach of relying solely on single boreholes to understand formation information, transforming the unknown into the known.
2. The cloud mapping and degradation zone classification model can identify low-resistivity anomaly areas, providing a basis for determining the presence of water conduits in the formations. However, by comparing the detection results with the results revealed by the detection borehole, there are some errors in the detection results. The reasons are analyzed to be affected by the measurement methods: (1) there is a deviation in the drilling hole, the arrangement of the cable and the spacing between the electrodes, etc., and the measurement accuracy still needs to be improved; (2) The influence of the hole spacing and hole depth of the method is large, so it has certain limitations. According to the field application, when the hole spacing: hole depth is greater than 1:2, there will be a large deviation in the measurement results; (3) This method can only detect the possible location of broken strata, but can’t know the actual broken degree of broken strata. The practice shows that the resistivity method can be used for water exploration in mine construction and shaft excavation.

## Conclusions

1. The resistivity of granite decreases with an increase in metallic mineral content, temperature, and joint angle. It also decreases with an increase in rock saturation and the ion content of water in fractures. For deep-seated formation detection techniques, a comparative analysis reveals that water content and joints (formation water content and degree of fracturing) are the main factors affecting resistivity.
2. During the loading degradation process, the resistivity of granite exhibits stage-responsive characteristics with respect to integrity, stress level, and damage accumulation. Under low-stress conditions, the rock is relatively intact with minimal internal damage, resulting in lower resistivity. As the stress level increases, the resistivity gradually decreases. However, once the stress level exceeds the threshold for crack initiation, the resistivity starts to increase. Finally, when the stress level reaches the peak stress and macroscopic cracks appear, a high-resistivity abrupt change occurs. Rock damage and the generation of macroscopic cracks are the main causes of resistivity abrupt changes.
3. Based on physical and mechanical test results, a degradation zone classification model is established to divide the formation degradation based on the resistivity baseline. In the presence of water, formations with resistivity above the baseline indicate relatively intact formations with low water content, while formations below the baseline indicate formations with poor integrity and high-water content. The opposite is true when there is no water. The zone degradation model provides a better description of the formation's occurrence state.
4. Resistivity measurements were conducted before and after grouting at the working face. By observing the changes in resistivity contour lines, low-resistivity anomaly areas can be preliminarily identified, and the water content of the formations can be determined preliminarily. By validating the degradation zone classification model, the degradation distribution of the formations in front of the working face before and after grouting can be further observed. This allows for a comprehensive evaluation and assessment of the grouting effectiveness between the formations.

## Data Availability

The datasets used and/or analysed during the current study available from the corresponding author on reasonable request.
